# Effects of Cigarette Smoke Condensate on Oxidative Stress, Apoptotic Cell Death, and HIV Replication in Human Monocytic Cells

**DOI:** 10.1371/journal.pone.0155791

**Published:** 2016-05-20

**Authors:** PSS Rao, Anusha Ande, Namita Sinha, Anil Kumar, Santosh Kumar

**Affiliations:** 1 Department of pharmaceutical sciences, College of Pharmacy, University of Tennessee Health Science Center, Memphis, Tennessee, United States of America; 2 Division of pharmacology and toxicology, School of Pharmacy, University of Missouri-Kansas City, Kansas City, Missouri, United States of America; H. Lee Moffitt Cancer Center & Research Institute, UNITED STATES

## Abstract

While cigarette smoking is prevalent amongst HIV-infected patients, the effects of cigarette smoke constituents in cells of myeloid lineage are poorly known. Recently, we have shown that nicotine induces oxidative stress through cytochrome P450 (CYP) 2A6-mediated pathway in U937 monocytic cells. The present study was designed to examine the effect of cigarette smoke condensate (CSC), which contains majority of tobacco constituents, on oxidative stress, cytotoxicity, expression of CYP1A1, and/or HIV-1 replication in HIV-infected (U1) and uninfected U937 cells. The effects of CSC on induction of CYP1 enzymes in HIV-infected primary macrophages were also analyzed. The results showed that the CSC-mediated increase in production of reactive oxygen species (ROS) in U937 cells is dose- and time-dependent. Moreover, CSC treatment was found to induce cytotoxicity in U937 cells through the apoptotic pathway via activation of caspase-3. Importantly, pretreatment with vitamin C blocked the CSC-mediated production of ROS and induction of caspase-3 activity. In U1 cells, acute treatment of CSC increased ROS production at 6H (>2-fold) and both ROS (>2 fold) and HIV-1 replication (>3-fold) after chronic treatment. The CSC mediated effects were associated with robust induction in the expression of CYP1A1 mRNA upon acute CSC treatment of U937 and U1 cells (>20-fold), and upon chronic CSC treatment to U1 cells (>30-fold). In addition, the CYP1A1 induction in U937 cells was mediated through the aromatic hydrocarbon receptor pathway. Lastly, CSC, which is known to increase viral replication in primary macrophages, was also found to induce CYP1 enzymes in HIV-infected primary macrophages. While mRNA levels of both CYP1A1 and CYP1B1 were elevated following CSC treatment, only CYP1B1 protein levels were increased in HIV-infected primary macrophages. In conclusion, these results suggest a possible association between oxidative stress, CYP1 expression, and viral replication in CSC-treated cells of myeloid lineage. This study warrants a closer examination of the role of CYP1B1 in smoking-mediated enhanced HIV replication.

## Introduction

Cigarette smoking is highly prevalent amongst people living with HIV/AIDS (PLWHA). A recent analysis of cross sectional surveys conducted in USA revealed that PLWHA were nearly twice as likely to smoke cigarette compared to the general population [1]. In addition to corroborating the high propensity of PLWHA towards cigarette smoking, the Centers for Disease Control and Prevention (CDC) and the U.S. Department of Health and Human Services estimate cigarette smoking to be responsible for loss of adherence to antiretroviral therapy (ART) and increased chances of acquiring secondary illnesses and infections in PLWHA [2, 3]. These adverse effects of cigarette smoking, along with the negative association of current smoking with learning, memory, and global cognitive function in PLWHA [4], have prompted the need and implementation of suitable intervention strategy for cigarette smoking cessation in PLWHA [5–7].

Given the high prevalence and adverse effects of cigarette smoking in PLWHA, it is critical to examine the impact of cigarette constituents on HIV replication. Our previous work has shown that HIV-infected smokers have a higher plasma viral load as compared to HIV-infected non-smokers [8]. Similarly, in vitro studies have reported an enhancement of HIV replication in cells subjected to cigarette/tobacco smoke [8–10]. However, the cellular pathways mediating the effects of cigarette constituents on HIV replication remain unclear.

In addition to its inherent carcinogenicity, cigarette smoke is a well-known inducer of oxidative stress. In monocytes/macrophages, which are known cellular target and reservoir for HIV infection [11, 12], exposure to cigarette smoke has been shown to disrupt the redox homeostasis [13], downregulate the expression of antioxidant genes [14, 15], and enhance the pro-inflammatory responses [16, 17]. Based on the significant role of oxidative stress in mediating HIV pathogenesis [18, 19], it is rationalized that exposure of myeloid lineage cells to cigarette constituents would result in enhanced oxidative stress and subsequent induction of cellular toxicity through apoptotic pathway as well as HIV replication in monocytic cells. We also propose that aromatic hydrocarbon receptor (AHR) -mediated induction of cytochrome P450 (CYP) is likely the possible mechanism for these effects.

To examine the effects of cigarette smoke condensate (CSC), which contains the majority of cigarette constituents, on oxidative stress and cytotoxicity, in this study, we utilized the human monocytic U937 cell line. Our recent studies have shown that nicotine, the major constituents of cigarette smoke, induces oxidative stress through CYP2A6-mediated metabolism nicotine in U937 as well as SVGA astrocytic cell lines [20, 21]. Further, to study the effects of CSC on HIV-1 replication, we used U1 monocytic cell line. U1 cell line is an HIV-infected U937 cell line which shows minimal constitutive expression of virus [22] and undergoes induction of viral expression upon differentiation into macrophages. These cells are considered the model system to study HIV-related effects in monocytes [23, 24]. Lastly, changes in expression of CYP1 enzymes, upon CSC treatment, were confirmed in HIV-infected primary macrophages.

## Materials and Methods

### Materials

The U937 monocytic cell line was purchased from American Type Culture Collection (ATCC, Manassas, VA). The HIV-infected U937 cell line—the U1 cell and the HIV-1Ada-M Monocytotropic Virus were obtained through the NIH AIDS Reagent Program (Germantown, MD). Buffy coats were purchased from local blood bank (Interstate Blood Bank Inc, Memphis, TN) and were supplied following screening for common infectious diseases like HIV and hepatitis B. Cell culture reagents including the Roswell Park Memorial Institute (RPMI) 1640 media, sodium bicarbonate solution, nonessential amino acid solution, and L-glutamine were purchased from Corning Inc. (Tewksbury, MA). Fetal bovine serum (heat-inactivated) was bought from Atlanta biologicals (Atlanta, GA). The recombinant Human IL-2 and recombinant Human macrophage colony stimulating factor (M-CSF) that were added to culture media of primary macrophages were obtained from R&D Systems and PeproTech, respectively. The RNeasy mini kit for collecting RNA and the AllPrep DNA/RNA/Protein Mini Kit were obtained from Qiagen (Valencia, CA). The Bicinchoninic Acid (BCA) protein assay kit, protease inhibitor cocktail, gene expression reagents, and primer probes (CYP1A1, Hs01054794_m1 and GAPDH) were obtained from Thermo Fisher Scientific (Waltham, MA). The Luminata crescendo western HRP substrate was purchased from EMD Millipore (Temecula, CA). Caspase-3 colorimetric assay kit for assessing caspase-3 activity was purchased from BioVision, Inc. (Milpitas, CA), and the HIV Type 1 p24 Antigen ELISA kit was obtained from ZeptoMetrix Corporation (Buffalo, NY). The 5-(and-6)-chloromethyl-2',7'-dichlorodihydrofluorescein diacetate, acetyl ester (CM-H2DCFDA) to detect intracellular reactive oxygen species (ROS) was purchased from Thermo Fisher Scientific (Waltham, MA). MTT salt and polybrene were bought from Sigma-Aldrich Co. (St. Louis, MO). The radioimmunoprecipitation assay (RIPA) buffer was obtained from Boston BioProducts, (Ashland, MA). Western blot set up and supplied were from Bio-Rad Laboratories, Inc (Hercules, CA). The AHR antagonist, CH223191, was purchased from Tocris Bioscience (Bristol, UK). Cigarette smoke condensate (CSC) was purchased from Murty Pharmaceuticals (Lexington KY). As per the manufacturer’s protocol, CSC was prepared by smoking University of Kentucky's 3R4F Standard Research Cigarettes on an FTC Smoke Machine. The Total Particulate Matter (TPM) on the filter was calculated by the weight gain of the filter. The condensate was extracted with DMSO by soaking and sonication to prepare a 4% (40 mg/mL) solution.

### Cell culture and treatment

To determine the immediate effects of CSC, monocytic cells (U937 and U1 cells) were incubated overnight at 37°C (0.4–0.8x10^6^ cells/ml) in RPMI media supplemented with 10% FBS and 1% L-glutamine. The media for U937 cells was additionally supplemented with nonessential amino acid solution, sodium bicarbonate, and Gentamycin while the media for U1 cells contained penicillin-streptomycin solution. Next day, cells were treated with CSC (50 μg/ml) or equivalent amount of vehicle for 3H (or mentioned time points) and collected for measuring the ROS production. Monocytic cells were pretreated with vitamin C, a known antioxidant, at a final concentration of 100 μM 1H before CSC treatment to block the ROS-mediated effects of CSC. Although higher doses of CSC (up to 200 μg/ml) have been used for in a previous in vitro study [25], to mimic physiologically relevant concentrations of cigarette constituents upon optimization a dose of 50 μg/ml was chosen for subsequent experiments [26, 27]. For the caspase-3 activity experiments, an additional dose of CSC, along with 1 ml of fresh media, was added 12H after the first treatment and the cells were collected at 24H. To determine the chronic effects of CSC treatment on HIV replication, a previously optimized protocol was used [8]. In brief, U1 cells were plate at 0.2x10^6^ cells/ml and treated once-daily with CSC (25 μg/ml) for 4-days. The cells were collected at 24H after the last dose, counted and plated in a 6-well plate at 0.6–0.8x10^6^ cells/2.5ml. Based on previously reported protocol [28], HIV-1 replication was induced by the addition of 100 nM phorbol-12-myristate-13-acetate (PMA) and supernatant was collected 24H after PMA addition to assess the concentration of HIV p24 antigen.

### Collection of peripheral blood mononuclear cells and HIV infection

Peripheral blood mononuclear cells (PBMCs) were collected from buffy coats following separation on density gradient using established protocol [29–31]. Briefly, the collected PBMCs were allowed to adhere to the plastic and the non-adherent cells were discarded after 4H. Adherent cells were cultured in media supplemented with M-CSF to facilitate macrophages differentiation and cells were collected after 7–10 days. The collected cells were treated with polybrene for 30 mins and infected with HIV-Ada strain at 20 ng/10^6^. The cells were seeded in 6-well plates and fresh media was added every third day to ensure cell viability. The p24 antigen levels were determined in collected culture supernatant using the p24 ELISA kit. Upon confirming viral infection (7–10 days), the HIV-infected primary macrophages were treated with vehicle (DMSO) or CSC (25 μg/mL), once-daily for four days. RNA and protein was collected 24H after the last treatment from the macrophages to determine changes in transcription and protein expression of CYP1 enzymes.

### Reactive oxygen species (ROS) production by flow cytometry

The level of reactive oxygen species (ROS) in vehicle- and CSC-treated monocytic cells was determined using the CM-H2DCFDA dye as described previously [32]. Briefly, following treatment with vehicle or CSC, monocytic cells were collected and washed with phosphate buffer saline (PBS). Subsequently, cells were incubated with the CM-H2DCFDA dye (2 to 5 μM) for 30 minutes at room temperature. After incubation, the cells were washed with PBS and the mean fluorescent intensity (MFI) for samples was measured using flow cytometer. The MFI obtained for vehicle-treated control cells served as 100%.

### MTT assay

Cell viability test was performed in U937 monocytes using the MTT assay [3-(4, 5-Dimethylthiazol-2-yl)-2, 5-diphenyltetrazolium bromide]. After 24H treatment with vehicle or CSC, cells were collected and washed with PBS. The cells were then incubated with 500 μl of 0.2 mg/ml MTT solution in fresh media for 4H. After incubation, the tubes were spun down and the formazan crystals formed were dissolved in 300 μl of DMSO. Subsequently, the absorbance was measured at 570/630 nm on Biorad Benchmark Plus microplate spectrophotometer (Biorad, Hercules, CA) using the Microplate Manager 5.2.1 software.

### Annexin V staining

To measure the effect of CSC treatment on apoptosis in U937 monocytes, the Annexin V apoptotic assay was performed. Briefly, after 48H treatment with CSC, cells were collected and resuspended in binding solution at a final concentration of 1 × 10^6^ cells per ml, 100 μl of which was transferred into a 5 ml tube. The cells were then incubated with 5 μl PE and 5 μl 7-AAD for 15 min at room temperature (in dark). For setting up compensation, the following controls were used: unstained, PE stained, 7-AAD stained tubes. After incubation, 400 μl binding solution was added to each tube and fluorescence was detected using a flow cytometer (BD Biosciences, San Jose, CA). The MFI was measured and analyzed.

### Caspase-3 cleavage activity

The caspase-3 activity in monocytic cells treated with CSC was determine using the caspase-3 colorimetric assay kit from BioVision, Inc. U937 cells were treated with 50 μg/mL CSC for the indicated time points. As per the kit protocol, the cytosolic extract from cell samples was collected following incubation with cell lysis buffer. Subsequently, the collected extract was incubated with the DEVD-pNA substrate and the cleavage of chromophore p-nitroaniline (pNA) from the labeled substrate was detected spectrophotometrically at 405 nm. The background reading from buffers was subtracted from the readings and the fold increase in caspase-3 activity was determined by normalizing against the activity obtained for vehicle-treated U937 cells. To inhibit the CSC-mediated induction of ROS, U937 cells were pretreated with vitamin C (100 μM) 1H before the addition of CSC, and the caspase-3 activity was measured at 24H.

### Western blot analysis

To determine the expression of CYP1A1 and CYP1B1 enzymes, equal amount of protein (20 μg) obtained from vehicle- and CSC-treated macrophages was loaded on a 10% polyacrylamide gel and electrophoresed, followed by transfer to a PVDF membrane. The membrane was blocked in 5% non-fat dry milk and incubated with primary antibody (1:500–1000 dilution) overnight. Next day, the membrane was washed and incubated with secondary antibody (1:2000 dilution). After washing, the blot was visualized by Luminata crescendo western HRP substrate using the Alpha Innotech FluorChem HD2 gel documentation system (Alpha Innotech, San Leandro, CA), and the densitometric data was analyzed using AlphaEase FC StandAlone software (version 6.0.0.14; Alpha Innotech). Glyceraldehyde-3-Phosphate Dehydrogenase (GAPDH) was used as an internal loading control to normalize the expression of CYP1A1 and CYP1B1 proteins.

### HIV Type 1 p24 ELISA

The Human Immunodeficiency Virus Type 1 (HIV-1) p24 antigen level in supernatant collected from U1 cells (24H post-PMA treatment) was determined using the enzyme linked immunoassay (HIV p24 ELISA). This assay is based on the principle that viral antigen in the media is specifically captured onto the immobilized monoclonal antibody for p24 coated on microwells. Following the manufacturer’s protocol, the captured viral antigen was sequentially exposed to a biotin-labeled human antibody to HIV-1, streptavidin conjugated to horseradish peroxidase, and tetramethylbenzidine substrate. The optical density of each well was measured at 450 nm using a microplate reader and compared against the standard curve to determine the concentration of HIV-1 p24 antigen (pg/ml) in the samples. The amount of HIV p24 from vehicle-treated U1 cells was treated as 100%.

### RTPCR

Quantitative RTPCR was performed to determine the transcription of CYP1A1 and CYP1B1 genes using previously described protocol [20]. Briefly, employing the 2-step TaqMan Gene Expression Kit (Thermo Fisher), 120 ng of RNA was reverse transcribed to cDNA and then amplified in the StepOnePlus system (Thermo Fisher). The relative fold expression for CYP1A1 gene in vehicle- and CSC-treated U937 cells (6H) and U1 cells (6H and 4-day) was calculated using 2^-ΔΔCt^ method by employing GAPDH as the housekeeping gene. Similar calculations were conducted to analyze the changes in transcription of CYP1A1 and CYP1B1 genes in HIV-infected primary macrophages.

### Statistical analysis

All experiments were conducted in triplicate. The experiment with PMA stimulation of U1 cells, following 4-day treatment with vehicle/CSC, was performed twice. The mean value obtained for vehicle-treated cells served as 100% for this study. The data obtained for CSC-treated cells was normalized against the control group and expressed as percent control. Student’s t-test was employed to analyze statistical differences (p≤0.05) between the vehicle- and CSC-treated monocytic cells. *p≤0.05; **p≤0.01

## Results

### CSC-induced ROS production in U937 cells

**Fig 1:**
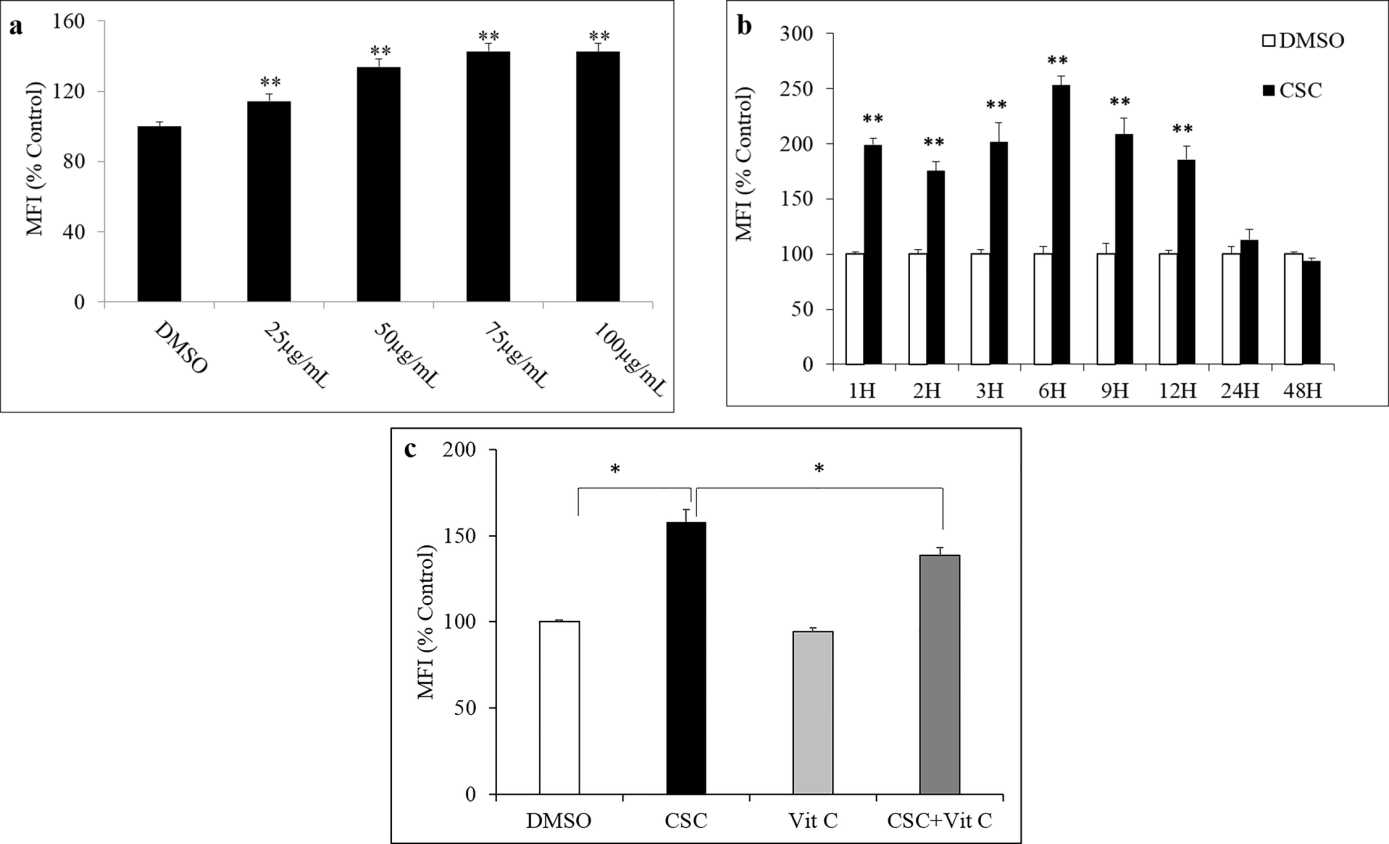
a) Dose-response of cigarette smoke condensate (CSC) treatment on reactive oxygen species (ROS) production in human U937 monocytic cells. CSC treatment at 3H was found to enhance ROS production in a dose-dependent manner. b) Time-kinetic of ROS induction following CSC treatment in U937 cells. CSC treatment (50 μg/ml) in U937 cells resulted in a time-dependent significant enhancement in ROS production as measured using a flow cytometer. Compared to the respective vehicle-treated cells, CSC-treated U937 cells were marked by significantly higher levels of ROS at 1H, 3H, 6H, 9H and 12H post-treatment. The peak for enhanced ROS production was 6H following CSC treatment. c) Effects of vitamin C pretreatment on induction of ROS production by CSC. Pretreatment with vitamin C significantly inhibited the CSC-mediated induction in ROS levels in U937 cells. *p≤0.05; **p≤0.01.

To measure the effects of CSC treatment on ROS levels in monocytic cells, U937 cells were treated with increasing concentration of CSC (25, 50, 70, and 100 μg/mL) for 3H. All tested concentrations of CSC were found to significantly enhance ROS levels in U937 cells (Fig 1A; p≤0.01). Based on this data, in subsequent experiments, 50 μg/mL was selected to evaluate the effects of CSC in monocytic cells. Treatment of U937 monocytic cells with 50 μg/ml CSC resulted in a time-dependent increase in production of ROS (Fig 1B). This increase in ROS levels, compared to vehicle-treated control cells, was significantly higher in CSC-treated U937 cells as early as 1H after 50 μg/ml CSC treatment and this effect on ROS levels lasted 12H post treatment (p≤0.01). The effect of CSC treatment on ROS production was minimal at extended time points (24H and 48H; p>0.05). Importantly, as depicted in Fig 1C, 1H pretreatment with vitamin C (100 μM) resulted in a significant reduction in CSC mediated (3H) induction of ROS levels in U937 cells (p≤0.05).

### CSC-induced caspase-3 activity in U937 cells

**Fig 2:**
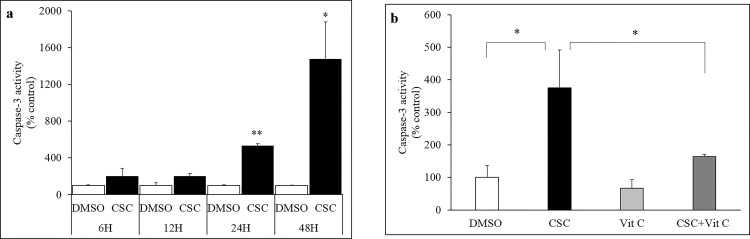
Caspase-3 activation in CSC treated U937 cells. a) Treatment with CSC was associated with a time-dependent increase in caspase-3 activity. The increase in caspase-3 activity, compared to vehicle-treated cells, was found to be significant 24H and 48H after CSC treatment. b) The CSC-mediated induction of caspase-3 activity was completely blocked following pretreatment with vitamin C in U937 cells. *p≤0.05; **p≤0.01.

To determine the downstream effects of CSC-mediated increase in ROS, the effect of CSC treatment on caspase-3 activity was determined in U937 cells. CSC treatment was found to be associated with a time-dependent enhancement of caspase-3 activity in U937 cells (Fig 2A). The induction in caspase-3 activity was found to be significant at 24H (p≤0.01) and 48H (p≤0.05) following CSC treatment. Moreover, 1H pretreatment with vitamin C (100 μM) significantly blocked the CSC-induced increase in caspase-3 activity in U937 cells (Fig 2B; p≤0.05).

### CSC-mediated cell death in U937 cells

**Fig 3:**
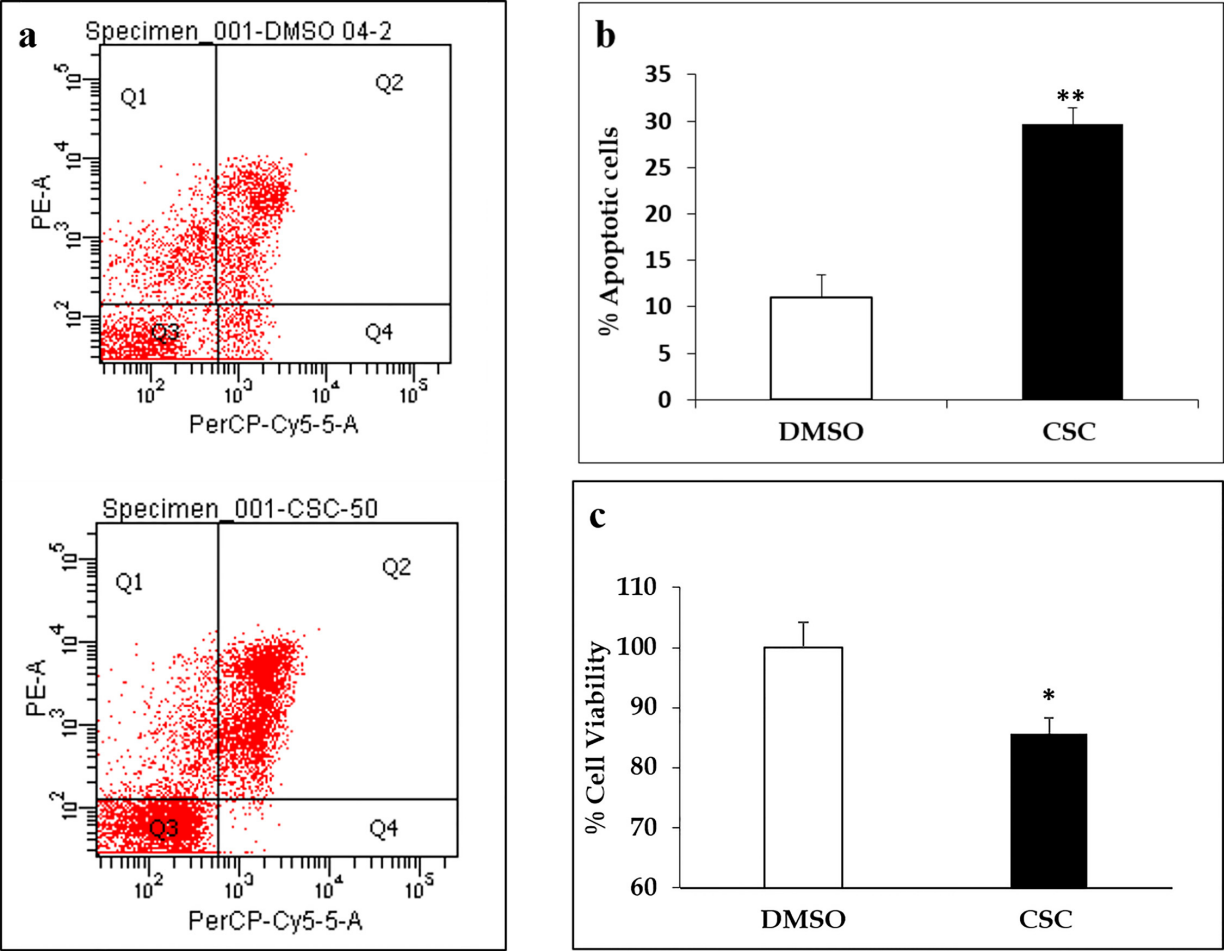
CSC treatment in U937 cells is associated with increased apoptosis. a) Representative dot plot shows that, compared to vehicle-treated control cells, a significantly higher proportion of U937 cells were positively stained with annexin dye following CSC treatment. b) Bar graph summarizing the percent apoptotic cells in Q2 from CSC treated U937 cells. c) Effect of CSC treatment on cell viability in U937 cells as measured by MTT assay. Results from MTT assay confirm a loss of cell viability in CSC treated U937 cells compared to vehicle-treated control cells. *p≤0.05; **p≤0.01.

The effects of CSC treatment on cell death in U937 monocytes were determined using Annexin V staining and MTT assay. U937 cells were treated with 50 μg/mL CSC for 48H and staining with Annexin reagent was performed to determine the apoptotic cell population. CSC treatment resulted in significant increase in apoptosis of monocytic cells compared to vehicle-treated control cells (Fig 3A). The bar graph (Fig 3B) represents percent apoptotic cells stained in Q2 from control and CSC-treated cells (p≤0.01). The CSC-mediated cell death in U937 cells was also confirmed using the MTT assay. As shown in Fig 3C, treatment with CSC (24H) resulted in significant loss of cell viability in U937 cells (p≤0.05).

### CSC-mediated production of ROS and HIV p24 upon chronic treatment in U1 cells

**Fig 4:**
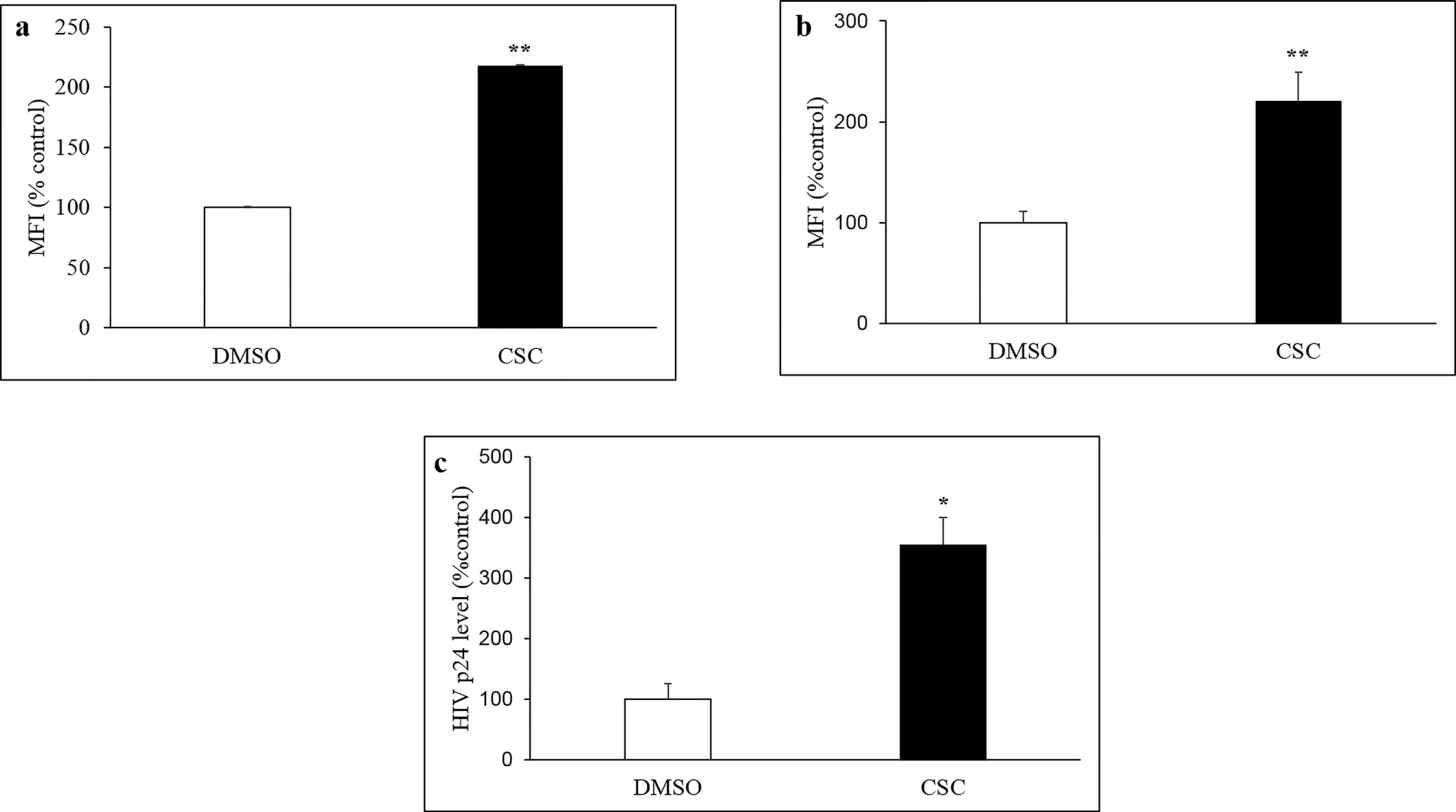
Effect of acute and chronic CSC treatment in HIV-infected U1 cells. a) CSC treatment (50 μg/ml) for 6H resulted in significantly enhanced level of ROS in U1 cells, compared to control cells. b) Chronic (4-day) daily treatment with CSC (25 μg/ml) resulted in significantly higher ROS levels in U1 cells. c) PMA stimulation following 4-day CSC treatment was associated with statistically significant increase in HIV replication, as measured by HIV-1 p24 antigen level in cell supernatant, in CSC treated U1 cells compared to vehicle-treated control group. *p≤0.05; **p≤0.01.

Upon studying the effects of CSC on the induction of ROS and apoptotic cell death in uninfected U937 cells, we studied the effect of CSC on ROS production and viral load (HIV p24) in HIV-infected U937 (U1) cells. Similar to U937 cells, the acute CSC (50μg/ml) treatment significantly induced (>2-fold) the ROS production at 6H in U1 cells compared to vehicle-treated control cells (Fig 4A; p≤0.01). Furthermore, the effect of chronic treatment with CSC (25 μg/ml) on ROS level and HIV p24 level in cell supernatant was also determined. CSC treatment (4-day) was associated with a significant enhancement in ROS production in U1 cells (Fig 4B; p≤0.01). Following PMA stimulation, the four-day, CSC-treated U1 cells were found to have significantly elevated HIV p24 level in the media, compared to vehicle-treated control cells, suggesting significantly elevated HIV replication following CSC treatment in U1 cells (Fig 4C; p≤0.05).

### CSC-mediated induction of CYP1A1 mRNA levels in U937 and U1 cells

**Fig 5:**
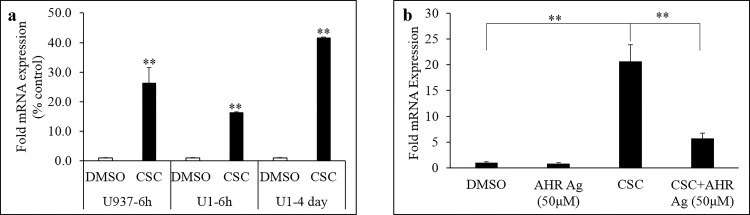
Effect of CSC treatment on CYP1A1 gene transcription in monocytic cells. a) mRNA levels of CYP1A1 were found to be significantly higher in CSC treated monocytic cells compared to control cells. Acute (6H) treatment of U937 and U1 cells with CSC resulted in ~25 and ~15 fold higher CYP1A1 mRNA level respectively, as compared to DMSO-treated cells. Chronic treatment (4-day) of U1 cells with CSC was also associated with statistically significant upregulation in CYP1A1 mRNA level (~40 fold). b) The CSC-mediated upregulation in CYP1A1 gene transcription was found to be mediated via the aromatic hydrocarbon receptor (AHR). Pretreatment of U937 cells with the AHR antagonist, CH223191, significantly blocked the CSC-mediated induction of CYP1A1 mRNA level. *p≤0.05; **p≤0.01.

To determine the possible associate between CYP1A1, the major polycyclic aromatic hydrocarbon (PAH)-metabolizing CYP enzymes, and CSC-mediated effects in monocytic cells, mRNA levels of CYP1A1 were assessed in vehicle- and CSC-treated U937 (6H) and U1 (6H and 4-day) cells. As indicated in Fig 5A, CSC-treated monocytic, uninfected U937, and HIV-infected U1 cells displayed a significant induction in transcription of CYP1A1. Compared to respective control cells, while the mRNA levels of CYP1A1 were induced by about 26-fold in U937 (6H treated), the mRNA induction was found to be about 16- and 42-fold in 6H and 4-day treated U1 cells, respectively. Furthermore, to confirm whether CSC-mediated CYP1A1 mRNA induction involves AHR signaling, U937 cells were pretreated with the AHR antagonist, CH223191, to block the nuclear translocation of AHR receptor. As summarized in Fig 5B, 1H pretreatment with CH223191 (50 μM) significantly blocked the CSC-mediated, after 3H, upregulation of CYP1A1 mRNA level in U937 cells (p≤0.01).

### CSC-mediated changes in expression of CYP1 enzymes in HIV-infected primary macrophages

**Fig 6:**
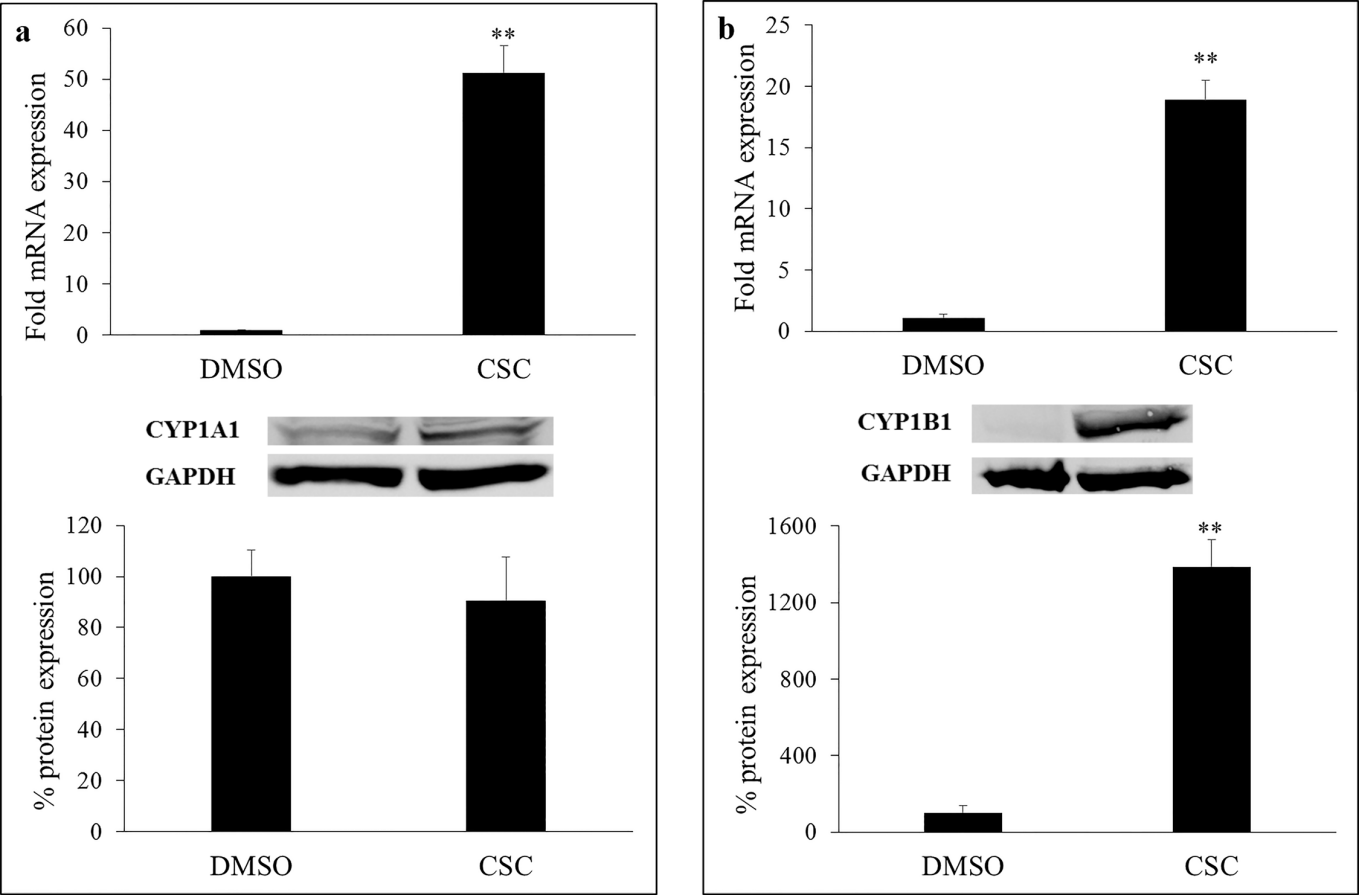
CSC-mediated changes in expression of CYP1 enzymes in HIV-infected human primary macrophages. a) Compared to the vehicle-treated control cells, CSC-treatment (4-day) was associated with an enhanced transcription of CYP1A1 gene in HIV-infected primary macrophages (upper panel). However, the expression of CYP1A1 protein was not upregulated following chronic treatment with CSC (lower panel). b) CSC-treatment (4-day) was associated with an enhanced transcription of CYP1B1 gene in HIV-infected primary macrophages (upper panel). CYP1B1 protein was also found to be significantly upregulated in CSC treated macrophages compared to vehicle treated HIV-infected human primary macrophages (lower panel). **p≤0.01.

While our previous study demonstrated CSC-mediated induction of viral replication in HIV-infected primary macrophages [8], CSC-mediated changes in transcription and protein expression of CYP1A1 and CYP1B1 in HIV-infected primary macrophages were determined in this study. As summarized in Fig 6A, 4-day CSC treatment in HIV-infected primary macrophages was associated with a significant induction of CYP1A1 gene transcription (top panel; p≤0.01). However, this increase in mRNA production did not translate in to induction of CYP1A1 protein expression in HIV-infected macrophages (p>0.05). This is not surprising because there are several reports including ours [32], in which, perhaps as a result of post-transcriptional modification or protein degradation protein induction does not match with the mRNA induction. Even though CYP1A1 protein is not induced by CSC, its basal level could be sufficient to metabolize tobacco constituents. CYP1B1 mRNA and protein expressions, on the other hand, were found to be significantly elevated in HIV-infected primary macrophages treated with CSC, compared to the vehicle-treated control cells (Fig 6B; p≤0.01). Representative Western blot image for CYP1A1, CYP1B1 and GAPDH (loading control) protein expression, along with the summarized bar graph results, summarize the changes associated with CSC treatment in HIV-infected primary macrophages.

## Discussion

**Fig 7:**
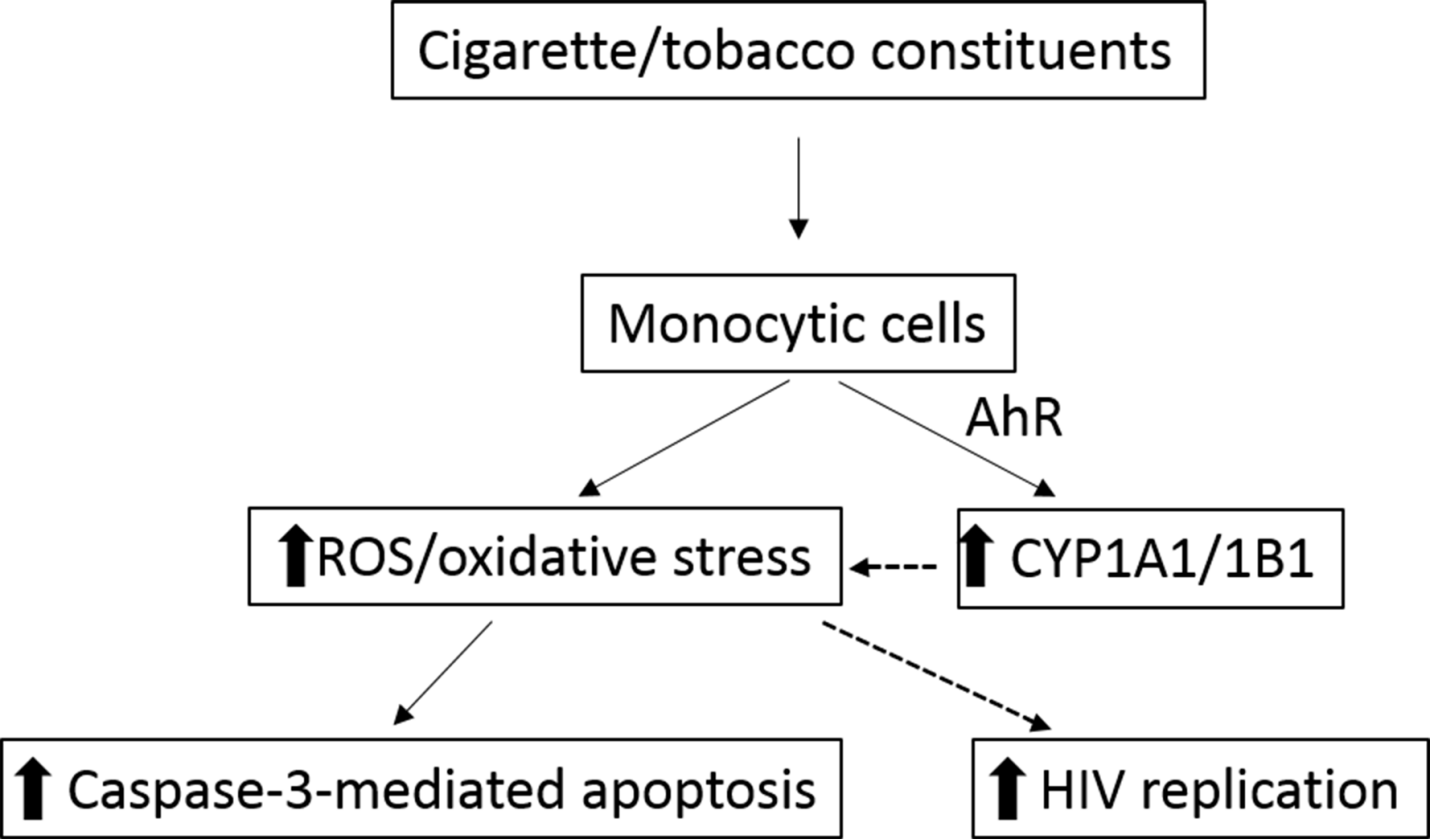
The rationalized cellular pathway for the effects of cigarette constituents/tobacco in monocytic cells. The AHR-mediated induction of CYP1 enzyme expression by cigarette constituents is likely to enhance ROS levels and oxidative stress. In addition, cigarette/tobacco constituents may directly increase oxidative stress through other pathways. Enhanced oxidative stress, in turn, is rationalized to activate the caspase-3 dependent apoptosis in monocytic cells. In HIV-infected macrophages, enhanced ROS production and oxidative stress are hypothesized to enhance viral replication, presumably through overexpression of CYP enzymes.

The results from this study provide direct evidence for CSC-mediated induction of ROS in HIV-infected and uninfected monocytic cells. The induction of ROS in U937 cells was observed to be dose- and time-dependent. Moreover, the increased level of ROS in U937 cells was associated with enhanced activation of caspase-3 activity and apoptosis in U937 cells. Importantly, pretreatment with antioxidant abolished the induction of ROS and caspase-3 activity in U937 cells, suggesting a direct role of ROS in CSC-induced caspase-3 activation. In U1 cells, a significant increase in ROS levels following chronic CSC treatment, compared to control cells, translated into an induction of HIV p24 levels assessed in the culture media 24H post PMA activation of U1 cells. This study, for the first time, has shown enhanced HIV replication in human monocytic U1 cells following CSC treatment. Moreover, the upregulation in ROS was concurrent with a significant enhancement in transcription of CYP1A1 gene in CSC-treated human monocytic cells, both U937 and U1 cells, compared to respective control cells. Lastly, we report a marked increase in expression of CYP1B1 enzyme in HIV-infected primary macrophages upon chronic treatment with CSC. Overall, as summarized in Fig 7, this study bolsters the possible involvement of cigarette constituent-mediated CYP1 induction and ROS generation (directly or through CYP-mediated activation of tobacco constituents) in apoptosis and cellular toxicity, as well as in the enhancement of HIV replication in monocytic cells.

Myeloid cells have been identified as critical targets for HIV and these cells are known to be much more persistent to the cytopathic effects of HIV, thereby serving as a reservoir for HIV infection [12, 22]. Monocytes, upon infection with HIV, are known to readily cross the blood-brain barrier [33] and are implicated in the development of neurological complications observed in HIV-infected patients [34, 35]. Therefore, uninfected U937 and HIV-infected U1 monocytic cell lines are important in vitro models to study the effects of cigarette/tobacco constituents and its implication on HIV infection. The U1 cell has been used extensively to study HIV pathogenesis [22] and to observe the effects of various treatments on post-PMA HIV replication [36], It serves as a prototypic model for direct assessment of the effects of cigarette constituents on HIV replication in monocytic cells. Our comprehensive recent studies have also utilized U937 as a monocytic cell model to determine the effects of nicotine and alcohol on oxidative stress and cellular toxicity and their underlying mechanisms through CYP-mediated pathway [21, 32, 37, 38]. Subsequently, we have been utilizing both U937 and U1 cells to study the effects of both alcohol and cigarette smoke constituents on HIV pathogenesis.

Cigarette smoke is a known inducer of oxidative damage in cells and this effect is primarily caused by the ROS and other free radicals that are present in cigarette smoke or formed intrinsically as a result of metabolism of cigarette constituents [39–41]. In addition to promoting oxidative damage, cigarette smoke-mediated ROS has also been implicated in inducing apoptosis in several cells including lings and liver [42]. However, the role of CSC in monocytic cell is poorly known. Herein, we report that CSC treatment in U937 cells is associated with a dose- and time-dependent induction of ROS production. The results from the present study also support CSC-mediated induction of ROS and subsequent apoptotic cell death in U937 monocytic cells. In fact, our results corroborate a previous finding wherein, increased level of ROS has been shown to induce caspase-3 activity [43]. Intuitively, pretreatment with the antioxidant vitamin C was found to significantly inhibit the CSC-induced production of ROS and subsequent activation of caspase-3 in U937 cells. However, the cellular pathways regulating ROS-induced activation of caspase-3, ER stress for instance [42, 43], needs to be further investigated. Given the prominent role of ROS-mediated caspase-3 activation in apoptosis [44], future studies will also assess the translation of enhanced caspase-3 activity, upon CSC treatment, in to programmed cell death in myeloid lineage cells.

Oxidative stress has been identified as a potent inducer of viral replication. Several markers of oxidative stress [45], including ROS [46, 47], are elevated during HIV infection and free radicals are known to further enhance HIV replication [18]. In fact, previous studies have delineated the cellular pathways responsible for free radical/ROS mediated induction in HIV replication. These studies have provided evidence for the involvement and activation of transcription factor, nuclear factor kappa-light-chain-enhancer of activated B cells (NFκB), in ROS-mediated enhancement of HIV replication [19]. Given the crucial role of NFκB in activating HIV replication [48] and the dynamics of increased ROS and HIV replication [18], it is not surprising that intracellular thiols which are responsible for neutralizing ROS have been found to control NFκB activation [49]. Hence, cellular changes in HIV-infected monocytes/macrophages leading to increased accumulation of ROS can be rationalized to directly influence the activation of NFκB and subsequently HIV replication. The present study furthers the current understanding of the interplay between oxidative stress and HIV replication by providing data for both enhanced ROS production and HIV replication in CSC treated U1 monocytic cells. Although the exact cellular pathway regulating HIV replication by CSC needs to be further studied, the existing literature on NFκB activation by CSC supports the possible involvement of NFκB activation in CSC-mediated induction of HIV replication in monocytic cells [50, 51].

The cytochrome P450 1 family includes three enzymes—CYP1A1, CYP1A2, and CYP1B1—and these enzymes are critical for the metabolism of the majority of xenobiotics, especially PAHs [52, 53]. In fact, CYP1A-mediated metabolism and activation of PAHs, commonly found in cigarette smoke, has been implicated in development of cancer [54]. The role of PAHs in enhancing HIV pathogenesis is bolstered by the fact that CYP1 enzyme catalyzed metabolic activation of PAH results in increased production of ROS and oxidative stress [55]. The CYP1A1 subtype is widely distributed in extrahepatic tissues and its expression is regulated by AHR [56]. Although the role of CYP1A1, CYP1A2, and CYP1B1 is well established in lungs and hepatic cells, their role is poorly known in extrahepatic cells, especially in monocytic cells. Interestingly, while CYP1B1 is minimally expressed, the basal level of CYP1A1 was found to be high in U937 monocytic cells, suggesting a potential role of CYP1A1 in these cells [32, 57]. In fact, the expression of CYP1 enzymes in the HIV-infected U1 cell remains undocumented till date. In this study, a robust induction in the expression of CYP1A1 in both U937 and U1 cells further suggest its role in monocytic cells. A correlation among CYP1A1 induction, oxidative stress, and HIV-1 replication corroborate with our hypothesis that CYP1 enzyme-induced oxidative stress is likely to promote HIV-1 replication in CSC treated monocytic cells. While our previous study demonstrated that CSC treatment increases HIV replication in primary macrophages [8], the data presented in this manuscript, for the first time, demonstrates significant induction in expression of CYP1B1 in CSC-treated, HIV-infected, primary macrophages. Further studies, however, are needed to establish the role of CYP-mediated oxidative stress pathway in CSC-induced enhancement of HIV-1 replication.

Our earlier study has shown that CYP2A6 is one of the most abundant CYP enzyme present in U937 and in primary monocytes [32]. Further, we have shown that CYP2A6 metabolizes nicotine into its primary metabolite, cotinine, and nitrogen-derived nitrosamine, as well as produces ROS in monocytic U937 as well as astrocytic SVGA cells [20, 21]. These findings were further verified using *ex vivo* study in primary monocytes obtained from smoker and HIV-infected smoker subjects [8, 58]. In these studies, HIV-infected smokers showed increased nicotine metabolism, oxidative stress, and HIV-1 replication compared with smoker or HIV+ subjects. However, the level of expression of CYP2A6 was not increased in smokers of HIV-infected smokers compared with their respective controls. This result was consistent with in vitro data, in which, CYP2A6 was not induced by nicotine in U937 cells. We speculated that, perhaps, the basal level of CYP2A6 is sufficient to metabolize nicotine and produce ROS leading to HIV-1 replication. In this context, a substantial increase in the level of CYP1A1 in both U937 and U1 cells suggests a potential role of CYP1A1 in PAH metabolism in monocytic cells leading to increased oxidative stress and perhaps viral replication. Similarly, it can be noted that nicotine increased only 15–20% of ROS production as opposed to CSC, which increased ROS production by >100%. Intuitively, enhanced expression of CYP1B1 can be rationalized to accelerate PAH metabolism in primary macrophages. Taken together, these results suggests a relatively higher contribution of PAHs compared to nicotine in oxidative stress and perhaps HIV-1 replication in monocytic cells. Further, it is imperative to study the relative contribution of individual PAHs, like benzo(a)pyrene, and their respective CYP enzymes in oxidative stress and HIV-1 replication in monocytic cells. Our future efforts are aimed towards examining the relative contribution of cigarette smoke constituents, especially PAHs, in oxidative stress-mediated HIV-1 replication, as well, as the mechanism by which these constituents increase HIV-1 replication. Specifically, given the abundance of CYP1B1 protein following CSC treatment, the role of CYP1 enzymes in CSC-mediated enhanced viral replication in HIV-infected primary macrophages will be assessed by modulating CYP1B1 expression by various inhibitors and activators. The outcomes from these studies would provide potentially novel targets to develop therapeutic interventions to treat HIV-infected smokers effectively.

## Supporting Information

S1 Dataset Supporting information that contains original data and/or statistical analysis for Figs 1–6. (XLSX)
